# Clinical Decision Support for Traumatic Brain Injury: Identifying a Framework for Practical Model-Based Intracranial Pressure Estimation at Multihour Timescales

**DOI:** 10.2196/23215

**Published:** 2021-03-22

**Authors:** J N Stroh, Tellen D Bennett, Vitaly Kheyfets, David Albers

**Affiliations:** 1 Department of Bioengineering University of Colorado Denver | Anschutz Medical Campus Aurora, CO United States; 2 Department of Pediatrics University of Colorado School of Medicine Aurora, CO United States

**Keywords:** intracranial pressure, traumatic brain injury, intracranial hypertension, patient-specific modeling, theoretical models

## Abstract

**Background:**

The clinical mitigation of intracranial hypertension due to traumatic brain injury requires timely knowledge of intracranial pressure to avoid secondary injury or death. Noninvasive intracranial pressure (nICP) estimation that operates sufficiently fast at multihour timescales and requires only common patient measurements is a desirable tool for clinical decision support and improving traumatic brain injury patient outcomes. However, existing model-based nICP estimation methods may be too slow or require data that are not easily obtained.

**Objective:**

This work considers short- and real-time nICP estimation at multihour timescales based on arterial blood pressure (ABP) to better inform the ongoing development of practical models with commonly available data.

**Methods:**

We assess and analyze the effects of two distinct pathways of model development, either by increasing physiological integration using a simple pressure estimation model, or by increasing physiological fidelity using a more complex model. Comparison of the model approaches is performed using a set of quantitative model validation criteria over hour-scale times applied to model nICP estimates in relation to observed ICP.

**Results:**

The simple fully coupled estimation scheme based on windowed regression outperforms a more complex nICP model with prescribed intracranial inflow when pulsatile ABP inflow conditions are provided. We also show that the simple estimation data requirements can be reduced to 1-minute averaged ABP summary data under generic waveform representation.

**Conclusions:**

Stronger performance of the simple bidirectional model indicates that feedback between the systemic vascular network and nICP estimation scheme is crucial for modeling over long intervals. However, simple model reduction to ABP-only dependence limits its utility in cases involving other brain injuries such as ischemic stroke and subarachnoid hemorrhage. Additional methodologies and considerations needed to overcome these limitations are illustrated and discussed.

## Introduction

### Background

Traumatic brain injury (TBI) is a major public health problem. Intracranial hypertension (ICH) is common after TBI and can cause secondary injury by decreasing local or global cerebral perfusion [1,2]. Cerebral autoregulation governs cerebral blood flow (CBF) by changing local artery diameter [3-5] and usually provides autonomic control of intracranial pressure (ICP). The capacity of this mechanism to adapt to pressure changes may be exhausted by sufficiently acute or prolonged hypertension, which can lead to insufficient perfusion following TBI. Impaired autoregulation also affects a patient’s response to drug therapies to reduce ICP [6]. Therefore, clinical management of ICH after brain injury is crucial for improving patient outcomes.

TBI is often accompanied by elevated systemic arterial blood pressure (ABP) and loss of cranial volume due to cerebral edema. The Monro-Kellie doctrine [7] postulates a constant volume of intracranial (IC) parenchyma (functional brain tissue) and fluids (blood and cerebrospinal fluid [CSF]), so changes in net fluid yield changes in ICP. Consequently, ABP is the primary external ICP driver under this hypothesis, together with changes in volume and fluid [8]. Therefore, clinical protocols seek to control ICP while maintaining cerebral perfusion pressure (CPP, the difference between ABP and ICP) [9] or risk cerebral hypoxia, which may result in death or permanent brain injury.

Important changes in patient ICP occur at minute-to-hour timescales, and clinicians need to know about them quickly. Decisions regarding the escalation of care and intervention for TBI patients are often driven by elevated ICP, typically defined as exceeding 20 mm Hg (1 mm Hg=133.3 Pa approximately) [10]. This underscores the need to monitor the ICP and identify critical changes. An ideal form of clinical decision support would predict ICP many minutes to a few hours in advance, as seconds or minutes might not provide adequate warning for timely intervention.

### The Need for ICP Estimation

ICP is measured in situ via an external ventricular drain (gold standard) or a fiberoptic intraparenchymal catheter. Both modalities are invasive and may adversely affect patient outcomes through the risks of infection and hemorrhage [11]. In some patients, the risks associated with monitoring are outweighed by the benefits of ICP- and CPP-guided therapy, but patient selection is critical. Alternatively, noninvasive intracranial pressure (nICP) estimation is less risky and could both inform patient selection and timing for monitor placement (eg, early for those who are predicted to benefit). It may also be paired with invasive ICP monitoring as a powerful clinical decision support tool. Methods of nICP estimation generally involve identifying relationships between ICP and proxies that may be more easily observable in real time. These relationships may be explored empirically or on the basis of explicit models representing underlying physiology; a recent comprehensive survey of nICP estimation modalities is available [12].

### Data and Clinical Availability

Estimation of ICP using models and/or proxy data is highly dependent on the availability of specific data, which limits its use. For example, nICP may be statistically estimated from ABP and concurrent measurements of CBF velocity or cerebral oxygenation via empirical relationships [13,14] or physiological models [15,16]. The collection of such data requires advanced techniques such as transcranial Doppler sonography or near-infrared spectroscopy, which are limited by the availability of instruments and trained technicians. These data must also typically undergo quality control, delaying their availability for nICP estimation. Although nICP may be estimated using various modalities, practical considerations such as clinical logistics and data timeliness render their applications difficult.

### TBI Modeling for Decision Support

An ideal model for clinical decision support of TBI management is one that quickly provides nICP forecasts at multihour timescales from commonly available data and includes IC (as an adjective) process resolution. Such a model does not currently exist. Fast methods based on machine learning and signal processing [17-21] provide empirical nICP forecasts but rely on an abundance of training and/or patient history data that may not be widely available. Real-time models that empirically approximate physiological relationships [15,16] are also fast, but they still require uncommon data and do not provide IC mechanism resolution useful for diagnosis or patient-specific tuning. Mechanistic modeling approaches [22-24] emphasize either broad systemic dynamics or short-time resolution of IC processes and may be too coarse or slow for the purpose of clinical nICP estimation.

Two recent models [15,16,23,24] have been cited extensively in this document. The more anatomically representative model of Ryu et al [23] estimates nICP from ABP without additional data but emphasizes pulse-scale pressure signals rather than hour-scale dynamics. The fast nICP estimation schemes of Kashif et al [15] track ICP at suitable multihour timescales but have stringent requirements for uncommon data, which limits their applicability. Although contrapuntal to one another, both models are foundational to this study, which focuses on the limits and extension of these methodologies for long-time nICP estimation from ABP.

### Objectives of This Paper

The different methodologies of Kashif et al [15] and Ryu et al [23] present two feasible options for nICP estimation: full systemic integration of the former’s simple model with a systemic hemodynamics model or unintegrated use of the more complex model of the latter. This investigation considers which model development strategy is a better initial step toward an ideal nICP estimation tool in a clinical setting. We present the advantages and disadvantages of each approach: to better inform the development of a tool representative of the ideal model, and to identify the input requirements for each model in relation to clinically available data.

This study has three primary objectives. The first is to extend the simplified nICP estimation framework [15,16] by using a coupled arterial vasculature model to eliminate its dependence on jointly measured CBF. The second is to evaluate the ABP-only simulation of this model and the one developed by Ryu et al [23] for nICP estimation over a duration of hours. The third goal is to clarify the additional model machinery, such as case-specific parameter estimation and inference, needed to implement nICP estimation for complex, clinically important situations. These goals aim to inform the development of a practical tool capable of providing timely support in the clinical decision-making process for TBI patients on a broader timescale than those considered in the literature.

The remainder of this paper is organized as follows: the *Methods* section presents the models and methods of investigation, describes the model experiments, and establishes the model assessment criterion; the *Results* section presents the results of the experiments and model comparison and discusses the simulations of cases involving other brain injuries such as ischemic stroke, which are poorly simulated without optimization; and the *Discussion* section summarizes the analysis and motivates ongoing work toward modeling nICP estimation in a particular direction on the basis of the results and implications.

## Methods

### Overview

The comparison of nICP estimation schemes involves three essential parts: model configurations, aortic inflow data that drive the system, and metrics used to compare models on the basis of various aspects of performance, which are presented in the following subsections.

### Numerical nICP Estimation Frameworks

#### Model Components

**Figure 1:**
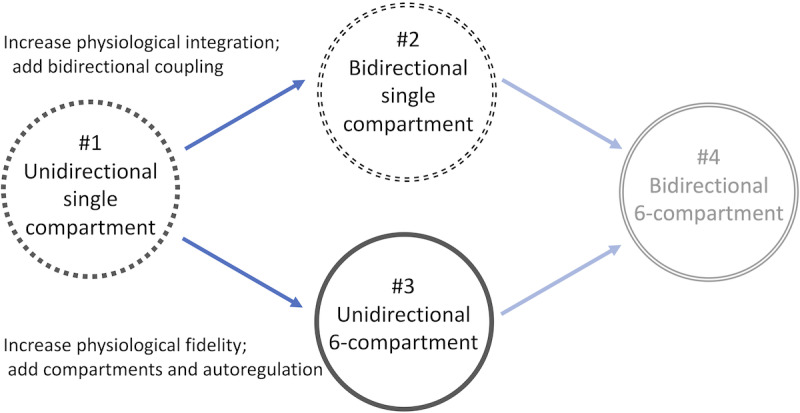
Conceptual overview of the relation among 4 models. The single-compartment model forced by prescribed hemodynamic time series (model #1) is the baseline model for comparison. Model #2 bidirectionally integrates the lower arterial network with the single-component intracranial model. In contrast, model #3 uses a more complex 6-compartment intracranial model with prescribed hemodynamic forcing. Model #4 represents of a multicompartment intracranial model fully integrated with the systemic arteries.

The models considered here are algorithms that transform aortic ABP data into nICP estimates using two components that may be coupled or independent. The first component is a vascular hemodynamics model that distributes ABP forcing through the systemic arterial network (AN) to the anatomical Circle of Willis (CoW), and is referred to as AN-CoW. The second component, referred to as the intracranial model (ICM), estimates nICP estimates using the outflow of the AN-CoW at the cranial arteries. We evaluated ICMs that either considered the cerebral perfusion system as a single compartment or as 6 interacting compartments defined by flow distributions of the anterior, middle, and posterior cerebral arteries. These compartments correspond to unresolved cerebrovascular territories perfused by the cerebral arteries [23], and these ICMs therefore differ in anatomical fidelity. The considered model formulations are differentiated by whether they interact unidirectionally or bidirectionally with the AN and by the complexity of the ICM component. The possible configurations are shown in Figure 1. In the unidirectional configurations, the AN-CoW boundary outflow at the middle cerebral artery (MCA) was prescribed to the ICM as an inflow boundary condition. The AN-CoW calculates this pressure and flow for the entire simulation, which is then applied to the ICM. Bidirectional coupling of the AN-CoW and ICM enforces interactive agreement of flow volumes and pressures at the interface of the components (enforced as conservation of current and voltage).

**Figure 2:**
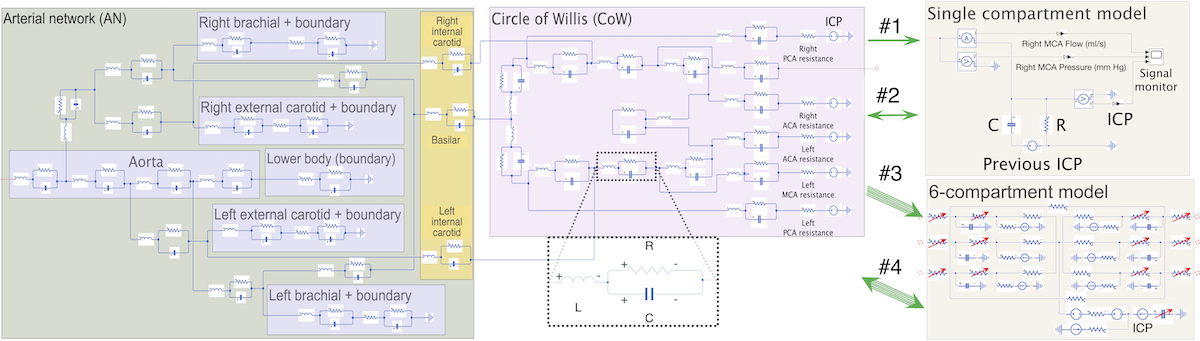
Diagram of model configurations 1–4. Schematic view of the various model configurations where green and pink boxes identify the AN and Circle of Willis vascular components, respectively, and intracranial models at right. Purple and orange boxes in the AN identify represented anatomy for reference. The vascular component is structured as in the source studies but uses 3-element electrical representations of each vessel, shown in the dashed white box. The single-compartment intracranial model is shown in the upper tan box; below it is a conceptual illustration of the 6-compartment model where red arrows indicate variable state components related to autoregulation and adaptive capacity. Unidirectional and bidirectional green arrows indicate the type of coupling between vascular and intracranial model components to distinguish configurations #1-4. ACA: anterior cerebral artery; ICP: intracranial pressure; MCA: middle cerebral artery; PCA: posterior cerebral artery.

Two directions for refining the base model are proposed as possible steps toward achieving a preferred but demanding model. Figure 1 shows the relationships of the models using model #1 as the most basic form and models #2 and #3 as parallel steps toward ideal model #4. Models #2 and #3 extend model #1 either by a bidirectionally coupled interface between the AN-CoW and ICM or by increasing the physiological complexity of the ICM component, respectively. This perspective also tests which choice yields the highest gain in improvement over model #1 and the cost of implementing it. Model #4 reflects the ultimate goal of a fully integrated bidirectional model featuring an anatomically accurate ICM. However, such a model is not presented here because of its difficult implementation and impractical computational cost for the simulation timescales considered. Bidirectional coupling is difficult for multicompartment models because of the codependency of the ICM state and the common pressure at each CoW terminal interface. Solving the ICM state equations at each time step requires several iterations, and each iterate requires recalculation of the entire upstream AN-CoW system constrained by pressure equality among the interfaces. The modeling framework used in this study is shown in Figure 2.

Each model comprises two separate model components, which are described below. The AN-CoW for resolving hemodynamics outside the cerebral territories is presented in the *Hemodynamical Modeling of Subcranial Arteries* subsection, whereas the ICMs for estimating ICP are presented in the *ICP Model Components* subsection.

#### Hemodynamical Modeling of Subcranial Arteries

Figure 2 depicts the AN-CoW model component, which comprises a subcranial AN (green box) and CoW vessels (pink box), as part of the modeling framework. As the spatial resolution of vessels is unnecessary, AN-CoW is modeled by a zero-dimensional framework of electrical analogs [25,26]. Each of the constituent 33 vessels was represented using a 3-element electrical analog (white inset box). This so-called lumped parameter approach has several advantages, including a relatively small number of patient-specific parameters. Furthermore, conservation laws at vessel interfaces reduce at each time step to algebraic systems rather than high-dimensional nonlinear functional representations [27] when spatially resolved.

**Figure 3:**
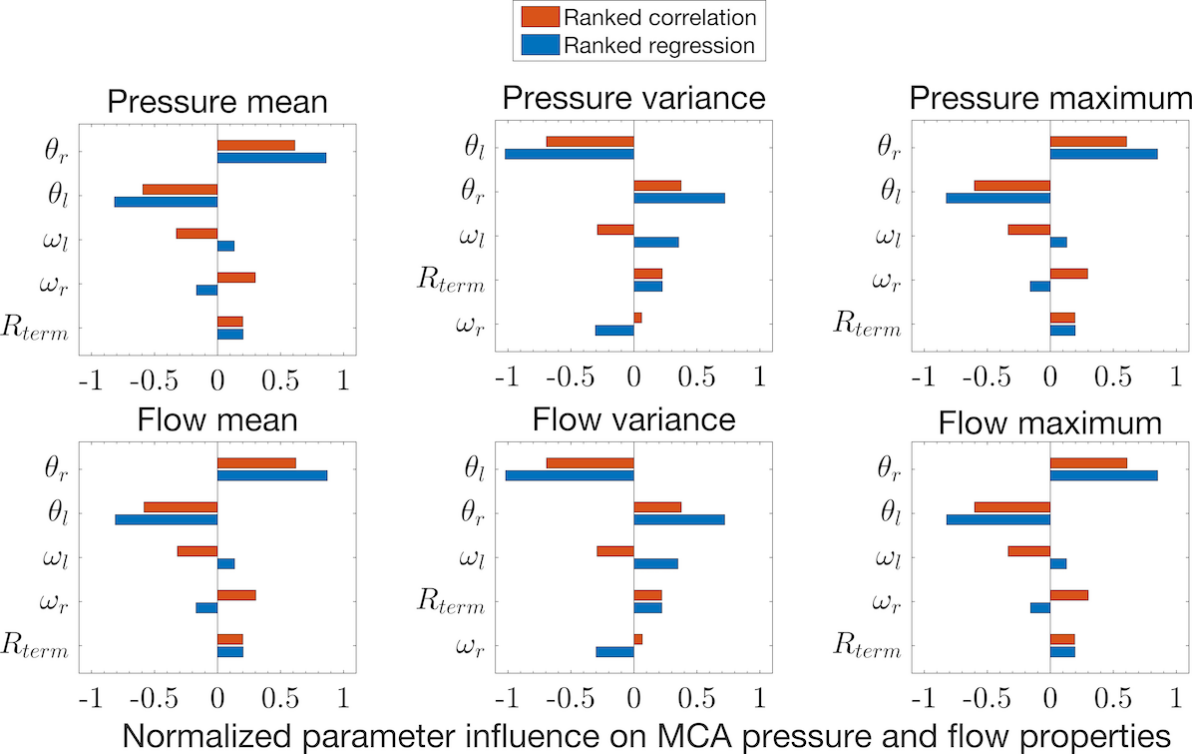
Ranked sensitivities of arterial network scaling parameters. Normalized empirical estimates of sensitivity ranking, shown here for key signal features (mean, variance, and maximum) of pressure (top row) and flow (bottom row) in the middle cerebral artery, summarize Monte Carlo experiments using global structured random uniform variations of scaling parameters (vertical axis of each panel). Parameter variations in vessel length (θ*_l_*) and radius (θ*_r_*) are most influential, whereas resistance scale (*R_term_*) and Windkessel scales (ω*_l_*, ω*_r_*) had relatively little impact on the solutions. The vessel dimension parameters have considerable influence on intracranial model inflow signals and provide global control while reducing the number of parameters needed to specify the hemodynamic model. MCA: middle cerebral artery.

Vascular network parameters total more than 100 but may be approximated by physically consistent functions of vessel length *l* and radius *r* [28]. A simple assumption of uniform dimensional scaling among the AN vessels is also applied to 3-element Windkessel boundaries and to the terminal resistances at CoW outflows. As CoW and adjacent vessel radii are approximately adult-sized by approximately 5 years of age [29], we did not scale vessels within the CoW model component. This reduces the large number of model parameters to only five effective parameters describing the scaling factors (proportions) of the base model values, which were adopted from a previous study [23] and references therein. This nonlinear reparametrization simplifies the AN-CoW component identification and is effective within realistic ranges of parameter values, as shown in Figure 3. Further details of the component definition, parametrization, boundaries, and sensitivity analysis are provided in Multimedia Appendix 1.

#### ICP Model Components

The ICM component is responsible for estimating nICP from the AN-CoW outflow to the cerebral arteries. The two ICM configurations considered are a 6-compartment model [23,30] and a single-compartment model [15,16], where each compartment represents a vascular perfusion territory. In addition to the number of represented cerebral perfusion territories, the models differ in their estimation approaches. The multicompartment model is more anatomically accurate and explicitly resolves IC hemodynamics with communicating arteries and autonomic pressure regulatory processes. In contrast, the single-compartment approach computes ICP using window-based statistical estimates of IC compliance and pressure determined through regression of the ICM inflow waveform properties. An overview of the multi- and single-compartment ICMs is presented in the following subsections.

#### Overview of the 6-Compartment Model

The complex model of Hu et al [30] and Ryu et al [23] presents an anatomical layout of the main cerebral pathways and their dependent mechanisms. Using six interacting territories, the model includes IC pressure and perfusion dynamics coupled by communicating arteries, dynamic autoregulation, and CSF balance. The autoregulatory processes are modeled as internal feedback mechanisms that regulate compartmental flow toward target values by controlling vessel radii [31]. This autonomic control influences the local pressure and flow balances between compartments, leading to intercompartmental blood flow via the communicating arteries. IC pressure and compliance are nonlinearly codetermined by volume changes resulting from autoregulation and net fluid change. The high degree of physiological fidelity resolves the IC dynamics at timescales inherited from ABP forcing. Furthermore, the 6-compartment nonlinear nICP component calculated numerous potentially clinically relevant diagnostic variables during the simulation. Unlike the source model, our implementation (model #3) is informed by the arterial inflow pressure and flow rate but does not provide feedback on systemic hemodynamics. A mathematical description, including a table of physiological and model parameters, is provided in Multimedia Appendix 2.

#### Overview of the Single-Compartment Model

The single-compartment ICM of Kashif et al [15] is a simple model that estimates ICP physiologically rather than anatomically modeling it. Here, nICP is constructed from linear regression estimates of bulk IC compliance (*C*) and resistance (*R*) over a temporal window containing several cardiac cycles. The algorithm estimates compliance *C* and resistance *R* by identifying the statistical relationships within a lumped parameter model representing IC physiology (details in Multimedia Appendix 3). These estimates and local ICP are related to MCA inflow and its applied pressure signal, from which nICP is deduced algebraically under the assumption of stationary parameter values.

The estimation process of this ICM requires no physiological parameters but requires algorithmic parameters that influence model behavior. Two required model hyper-parameters control the length of the temporal window over which each estimation occurs and the time step of the parameter updates. The first is limited by the stationarity assumption and determines the sample size for the regressions, whereas the second controls the output temporal resolution and coupling strength. Under bidirectional coupling, our implementation defines nICP as the simulated forecast based on the previous values of nICP and resistance *R*. These latter quantities were fixed in unidirectional coupling setups. Therefore, the length of the update time step affects the temporal coarseness of the nICP estimate in each model and defines the timescale of feedback between the ICM and upstream vascular model in the bidirectional model. Single-compartment model simulations use 1-minute windows and 1-minute updates, unless otherwise specified.

### Observational Data and Patient Selection

The CHARIS v1.0.0 collection (Charis hereafter [32]), publicly available from PhysioNet [33], comprises 50 Hz joint radial ABP and ICP time series of 13 patients. These data satisfy the model requirements, including documentation of diagnosed IC injuries, and suffice for model input and evaluation. Using radial ABP data as aortic introduces biases against systolic pressure more than diastolic [34,35], and these errors are consistent among our experiments. Sophisticated transformations exist [36] for reconstructing aortic pressure from radial ABP, but the simple approach taken here avoids uncertainties associated with additional algorithmic processing.

For model comparison, this study focuses on Charis patient #6, a 20-year-old male with TBI, based on the simplicity of his injury, cleanliness of joint ABP-ICP signal, and representativeness of base parameters (eg, optimal scaling parameters for the AN-CoW were approximately 1). In addition, large-scale noise or corrupt signals are common in the records of the patients (Multimedia Appendix 4); most of their ABP and/or ICP data could not be used contiguously for 4- to 6-hour periods without extensive and uncertain preprocessing of the available data.

**Figure 4:**
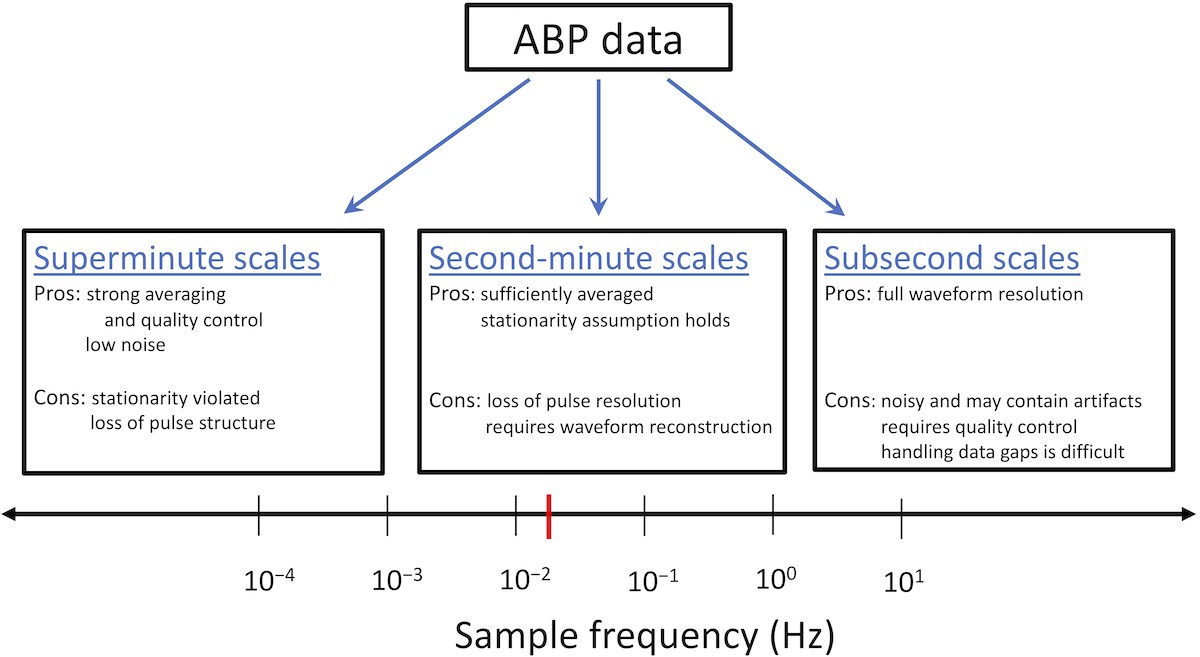
Timescales of ABP inflow data. The complex models can run on data from any part of the sampling spectrum. Simple models require pulsatile inflow from the rightmost portion of the scale (above about 10 Hz), which may not be typically available. The central scale is desirable for hour-scale applications, as this resolution both qualitatively minimizes computational overhead and supports parameter stationarity assumed in the regressive single-compartment models. The quaque 1-min data sampling frequency is indicated in red. The left-most scale offers strong smoothing and low noise but fails to resolve pulsatile waveform and violates assumptions of the simple models. ABP: arterial blood pressure.

Figure 4 identifies the possible sampling frequencies for the aortic model inflow. Models #1 and #2 have stricter aortic inflow requirements than models #3 and #4, as their simpler ICMs require ABP sample frequency in the rightmost portion of the scale (<10 Hz) for waveform feature identification. For example, previous studies [15,16] validated the regressive method of the simple ICM using data sampled at 20-70 Hz and 125 Hz. Such data are obtainable but are not commonly available and typically require quality control.

Lower-frequency ABP time series, which are more accessible and cleaner, are assumed to comprise nonoverlapping 1-minute (quaque 1-minute [q1m]) averages of systolic and diastolic pressures and heart rate. A waveform model (defined by a superposition of beta distribution probability density functions; Multimedia Appendix 5) projects these discrete q1m data into continuous time using patient-specific waveform parameters 𝛾, which are then sampled for convenience at 60 Hz. In relation to Figure 4, this process maps q1m data (identified by the red mark) into the scale usable by the simpler models to test the robustness of their data requirements.

### Measures of Quality and Efficiency for Models

Each experiment was evaluated using three scores: rating error, classification accuracy, and speed for simulations over time interval [*0,T*] in *N* 1-minute intervals, which quantify the desirable properties of the nICP estimates [37,38] for the purposes of relative comparison. The symbol nICP^*^ herein indicates nICP debiased against the observed ICP during the first hour of the simulation. The justification for this correction is that skill scores evaluate the model’s ability to track variability in recorded ICP data rather than estimate the absolute pressure. It also accounts for some of the bias introduced through the misuse of radial blood pressure as aortic inflow pressure. Each evaluation is applied to an nICP estimate, the score of which is then associated with the model that produces it.

The first score is the time-averaged standard error between the ICP and the debiased model estimate:

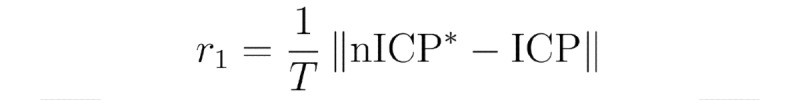



This rates the ability of the model nICP to track the observed ICP changes and quantifies the general inaccuracy of the model nICP estimate in observed units. Scaling by the simulation length allows comparison over different simulation lengths.

The second evaluation is the mean percentage of time that nICP correctly agrees with the observed criticality (ICP>20 mm Hg) during the total *N*-minute simulation. The measure of model accuracy is defined as:

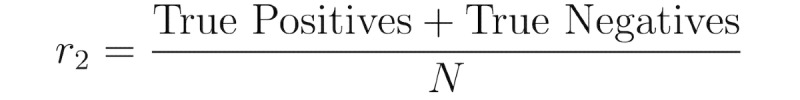



Although more qualitative than *r*_1_, classification accuracy may be more relevant for clinical decision support as it quantifies the coherence between the model and observed critical ICP [39].

Finally, the third quantity is simply the ratio of the simulated time interval to the elapsed clock time:

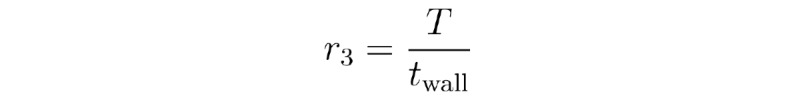



with *r*_3_>1 indicating a faster-than-real-time forward model integration. The values of *t_wall_* correspond to serial run times using MATLAB R2020a with a 3.7 GHz Intel i5 central processing unit. This final evaluation measures the practicality of a model for providing timely clinical support as well as its utility in other applications, such as nonlinear parameter estimation or data assimilation methods that require extensive, repeated model simulation.

The number of necessary parameters required for realistic initialization and the input data fidelity were assessed in the context of model utility, but they were not evaluated quantitatively. Finally, all model simulations are initialized with zero flow within the AN-CoW system common to the various model configurations. A spin-up adjustment occurs in the first 2 to 3 minutes of simulation, and these errors are included in the skill calculation with negligible impact on comparative assessment.

## Results

### Comparative Assessment of Model Simulations

**Figure 5:**
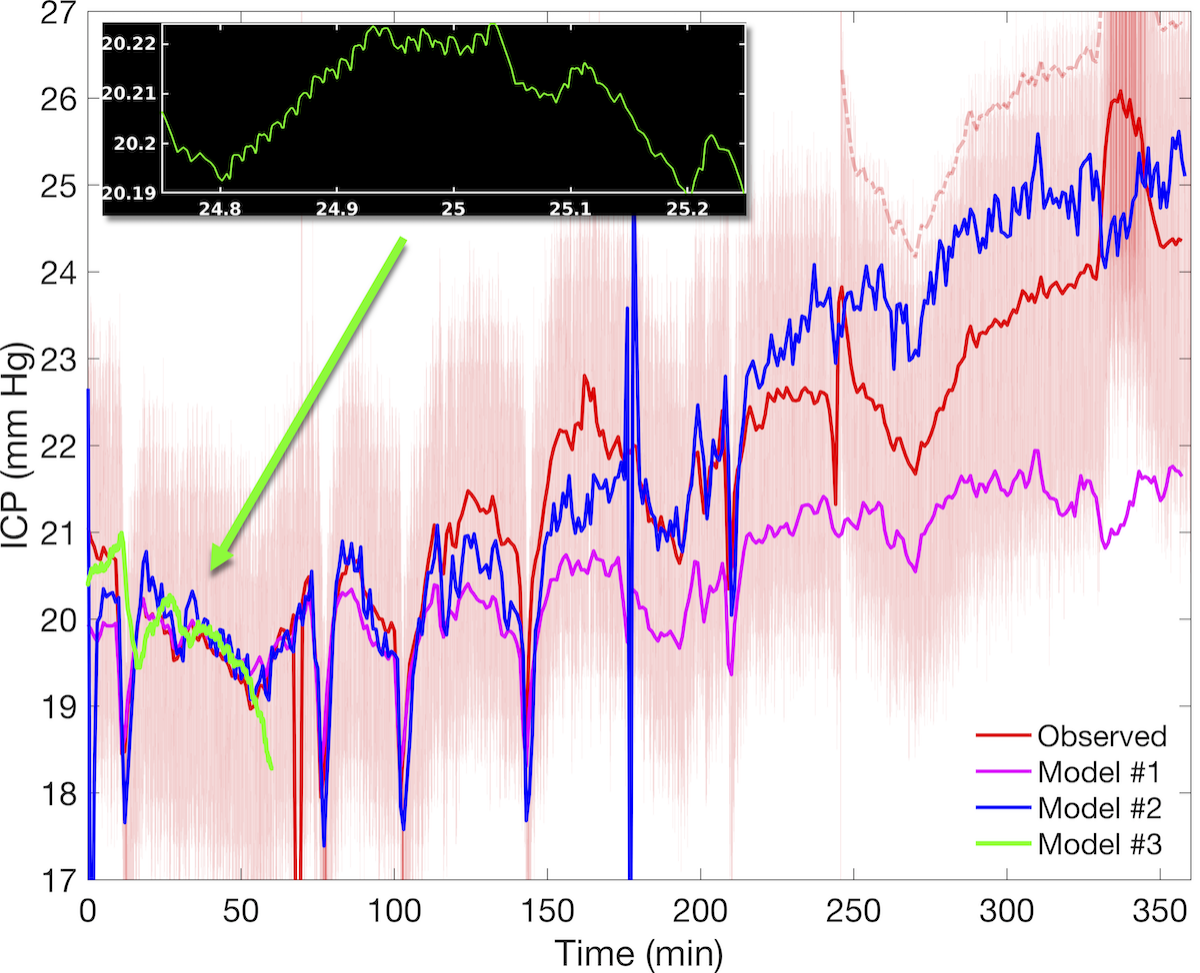
Observed and estimated noninvasive ICP for patient #6. Depicted are the observed (red) and estimated noninvasive ICP for Charis patient #6 using models #1-3, with model #2 showing the best accuracy. The noninvasive ICP estimated by model #1 (magenta) requires less than 5 minutes to run but has larger long-term errors. Model #2 (blue) takes approximately 45 minutes but produces a more accurate noninvasive ICP trend. Model #3 (green) estimates 1 hour of noninvasive ICP in approximately 6 hours of clock time; it requires variance inflation to obtain the curve shown. Model biases over the first hour are approximately 6.5 mm Hg, excluding spin-up errors. The black inset illustrates model #3 pulse resolution during a 30-second interval. Bidirectionality in model #2 has better low-frequency resolution and trend tracking than model #1, but makes it susceptible to feedback-driven instability under noisy inflow data (models #1 and #2 near 180 minutes). ICP: intracranial pressure.

Assessment of nICP and model efficiency for the first hours of patient #6 indicates that model #2 has a lower error than model #1 and is more practical than model #3. Figure 5 shows the observed ICP signal along with the estimates from models #1 to #3. Patient ICP was initially stable near 20 mm Hg for approximately an hour and gradually increased to approximately 24 mm Hg during the final 2.5 hours. Temporary pressure drops near 10, 75, 105, and 142 minutes likely reflect interventions (eg, mannitol or hyperventilation treatments) [32]. The observed ICP signal used in the model evaluation is shown in solid red. This reference ICP is decreased by approximately 2.5 mm Hg after 243.5 minutes to compensate for the sharp 5+ mm Hg record discontinuity, which may be due to transducer recalibration. The original unaltered 1-minute average ICP observations (dashed light red) are shown for reference over the interval 244 to 360 minutes.

Model comparison is organized into three subtopics: qualitative differences, quantitative differences, and observations about resolvable timescales and fidelity.

#### Qualitative Differences Between nICP Series

Models #1 and #2 produce qualitatively different pressure estimates, with the key difference being that model #2 follows the multihour trend of increasing ICP. Model #1 tracks the observations well for approximately 2 hours but fails to track the subsequent ICP elevation, as its bias falls from −1.8 mm Hg to nearly −3.2 mm Hg during 220 to 360 minutes. Model #2 tracks this observed pressure rise, although there is a roughly uniform bias of 1.02 mm Hg during this same period. One concludes that feedback from bidirectional coupling improves the estimation of low-frequency ICP signal components that are crucial in applications spanning several hours. Note that the observed 2 mm Hg pressure event (330-350 minutes) was resolved by neither model. This feature may be the result of a temporary change in patient posture, but no corresponding change occurs in the aortic ABP inflow signal (Multimedia Appendix 4, center left panel). This provides evidence that changes in ICP that do not arise from aortic ABP dynamics may not be resolved by simple ICMs.

The poorly identified parameters and long computation time hindered the simulation of model #3 for longer than 1 hour. The default ICM parameters [23] did not generate realistic ICP and required alteration of venous capillary conductance (*G_pv_)* and reference pressure (*P_icn_*) to obtain the reported nICP estimate. Small exploratory changes in parameter values often led to nICP divergence, indicating a strong dynamical dependence on parameters that must be inferred before useful simulation. The reported solution also includes a mean variance inflation of 26.3, which compensates for uncalibrated parameters, although the localized pulse amplitude (Figure 5, inset) is still too weak. This modified nICP estimates the observed trend well, although it lags behind the observations by approximately 4 minutes. This apparent delay, such as the reduced variance at several timescales, likely reflects poor representation by generic ICM parameters in the absence of additional inference. Attempts to determine more accurate parameter values were limited by model speed, which is approximately 6 times slower than real time.

#### Quantitative Differences Between nICP Series

The qualitative advantages of the bidirectional simple model over the unidirectional complex model are borne out by model skills *r*_1_-*r*_3_, as shown in Table 1. Further classification metrics for this case are given in Multimedia Appendix 6. Scores for the commonly resolved first hour appear in parentheses to account for differences in the simulation period. Bidirectional coupling reduces the simple model error from approximately 5 mm Hg to approximately 3.5 mm Hg (an improvement of nearly 30%), whereas critical ICP is estimated more accurately by 9.3 percentage points (a 10.6% relative improvement). Over the first hour, there was a slight increase in model #2 error due to longer spin-up adjustment and a modest 6 percentage point improvement in critical ICP detection. The complex unidirectionally coupled model #3 shows less than 1% improvement in critical ICP identification over the base model, with mean error increasing to 3.83 mm Hg (a 57% increase relative to model #1) mostly due to the approximately 4-minute lag. Accounting for this delay reduces model #3 error to 2.75 mm Hg but also reduces accuracy to *r*_2_=0.84; this affects neither skill ranking of model #2 over model #3. These results support that the feedback mechanism improves low-frequency tracking, which has little advantage over short timescales, and also suggests that model #2 has a practical advantage over model #3 in terms of error and accuracy.

**Table 1:** Model scores for principal comparison.^a^

Model	Error: *r*_1_ 6 hour (first hour)	Accuracy: *r*_2_ 6 hour (first hour)	Speed: *r*_3_ 6 hour (first hour)
Model #1	5.01 (2.42)	0.877 (0.92)	*116.129* ^b^
Model #2	*3.53* (2.47)	*0.97* (0.98)	7.356
Model #3	(3.83)	(0.883)	(0.145)

^a^Scores for simulations of Charis patient #6 during initial hours of data. Scores *r*_1_ and *r*_2_ rate the nICP errors and accuracy in identifying critical ICP, respectively, whereas score *r*_3_ rates the speed of the nICP estimation process. Parenthesized entries are calculated using only the first simulated hour.

^b^Italic text indicates the best results for each score.

The most significant difference between models #2 and #3 for practical nICP estimation is in the simulation speed (measured by *r*_3_). Both models #1 and #2 operate considerably faster than real time and are therefore suitable for an operational clinical support system. Model #3, however, is an order of magnitude slower than wall time under the same forcing and ill-suited for multihour simulation under pulsatile forcing. The speed of model #3 is limited by the calculation of many (*O*(10^3^)) iterative solutions to its nonlinear ICM per cardiac cycle, primarily during systolic upswing. A previous study using this ICM [24] reported that each cardiac cycle required 40 seconds within their highly optimized numerical framework. As their implementation used a one-dimensional AN-CoW, the speed of model #4 had a lower bound of *r*_3_=0.0225.

#### Resolution Versus Speed Considerations

**Figure 6:**
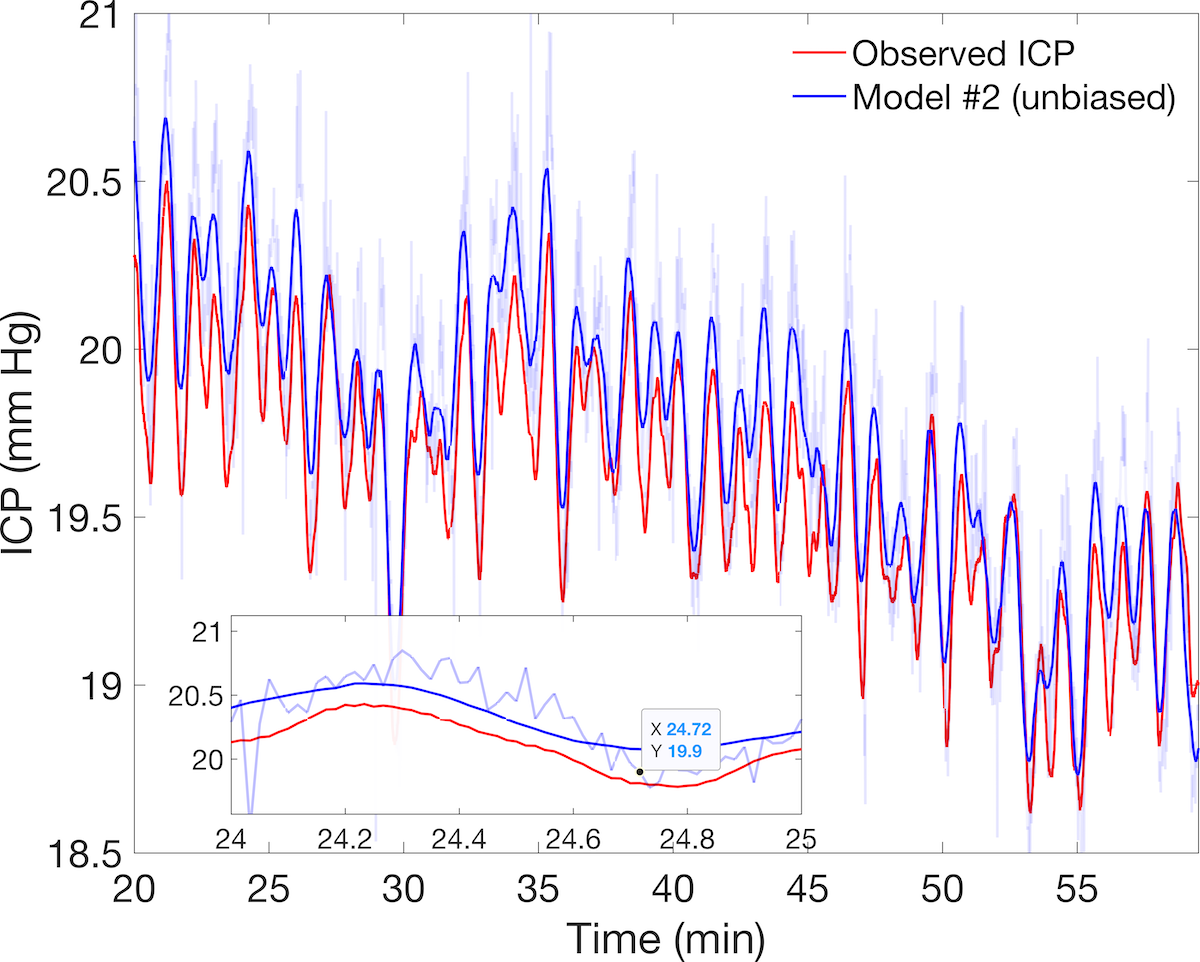
Strong local tracking of the ICP signal in model #2 at the expense of computational time. The mean noninvasive intracranial pressure estimates over 30-second intervals (blue curve) using the output of model #2 (light blue) with raw arterial blood pressure strongly track the observed ICP (red curve). The model simulation accurately reproduces local trends and *O*(10^−2^) Hz waves of the averaged observed ICP. This simulation calculated resistance and compliance parameters at 1-second intervals using a 30-second moving window (ie, with a 29-second overlap). The corresponding mean ICP estimates are plotted as solid curves for comparison with the observed ICP, with an inset showing the lack of subminute resolution. Although 4 times slower than real time, this simulation is roughly twice as fast as model #3 under pulsatile aortic inflow and requires no additional data or external inference. ICP: intracranial pressure.

Models also differ in their ABP data requirements, and one must consider the trade-off between the desired nICP temporal resolution and model efficiency. The complex ICM is defined by differential equations, so fine timescales inherited from pulsatile inflow boundary conditions require extensive, inflexible computation time to resolve the nICP pulse (black inset, Figure 5). The use of q1m mean ABP inflow increases model #3 speed considerably to *r*_3_=1.15 (slightly faster than real time), and analysis suggests this should not impair the resolution of autoregulation effects, which manifest at timescales beyond 15 seconds. On the other hand, simple model hyper-parameters (window length and parameter update interval) can be adjusted to resolve higher-frequency nICP components with additional computational time. Figure 6 illustrates a model #2 simulation under raw ABP using a 30-second window and 1-second update period (ie, 29 second overlap). Additional computational overhead reduces speed (*r*_3_=0.25 approximately), but there is considerable gain in nICP fidelity at high frequencies as well as strongly reduced error (*r*_1_) and increased accuracy (*r*_2_). This demonstrates the latent ability of model #2 to estimate higher-frequency components of ICP from ABP without additional ICM parameter inference, as in model #3.

### Simple Model Experiments With Low-Frequency Inflow Data

**Figure 7:**
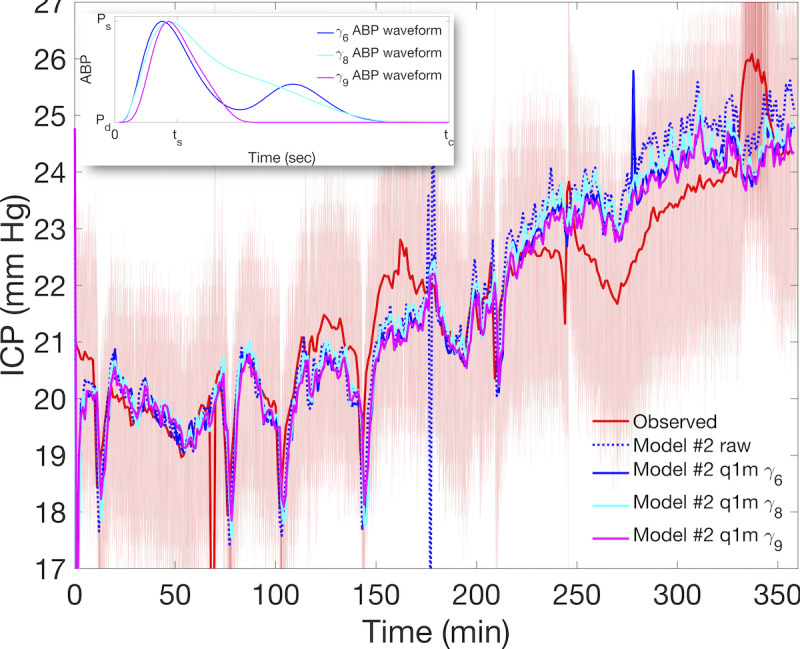
Model #2 performance using quaque 1-min (q1m) summary arterial blood pressure (ABP) data for Charis patient #6. Various simulations using q1m inflow data are compared with observed intracranial pressure (ICP; red curve) and noninvasive intracranial pressure (nICP) estimate based on raw 50 Hz data (dashed blue). Also shown are estimates using minute-wise constant continuous representatives of q1m ABP data generated by correct (blue) and incorrect (magenta and cyan) waveform parameters. The figure inset shows ABP waveform shapes for patients #6 (solid blue), #8 (cyan), and #9 (magenta), respectively, which yield qualitatively indistinguishable nICP estimates in the main plot. This shows that q1m ABP is sufficient for the aortic inflow and that patient-specific parametrization of ABP waveforms has little advantage in the simple model. ICP: intracranial pressure; P_s_: systolic pressure; P_d_: diastolic pressure; t_s_: systolic upswing duration; t_c_: cardiac cycle time.

Models can use commonly available ABP summary records under appropriate representation without additional waveform data. The use of models #1 and #2 is limited by pulse-resolving ABP inflow, but this requirement may be weakened using waveform transformation of q1m ABP summary time series of phase pressures and heart rate (Multimedia Appendix 5). Figure 7 shows that the original nICP estimate of model #2 (blue dashed) and one using q1m inflow (*γ*_6_, solid blue) are largely indistinguishable, although smoother inflow data of the latter avoids the instability around 175 minutes. The estimate from q1m ABP has a 3% larger error (*r*_1_=3.7 mm Hg), although there is no qualitative difference in clinical accuracy or speed compared with the original estimate. Furthermore, the lack of patient-specific waveform parameters has little effect on simulated nICP in this model: all model scores are roughly preserved in 2 additional runs (cyan and magenta) using waveform parameters of Charis patients #8 and #9 (*γ*_8_ and *γ*_9_, respectively), which differ in postsystole shape (inset). However, model #2 nICP estimates based on q1m ABP without heart rate data (not shown) were highly inaccurate due to errors in the numerical calculation of the ICM inflow pressure derivative.

### Summary of Assessments and Experiments

#### Strengths and Weaknesses of the Bidirectionally Coupled Simple Model Approach

The model comparison suggests that bidirectional coupling strengthens the resolution of low-frequency nICP trends, which are crucial in multihour simulations, and improves the critical nICP classification accuracy by approximately 10%. Temporal estimation of *O*(10^−2^) Hz ICP features is possible with no additional ICM parameter inference but requires additional computation time. Bidirectional coupling makes the model more prone to potential instabilities during spin-up and in the presence of noisy ABP inflow data. Using waveform projections of q1m summary ABP data as inflow data neither decreases nICP estimate quality nor requires patient-specific waveform parameterization, which both broadens applicability and decreases inflow noise. The simple model framework is still limited by its lack of internal process resolution and primarily responds to temporal variations in applied aortic inflow, but the fully coupled simple model approach is an order of magnitude faster than the clock time. Therefore, it has sufficient computational headroom to incorporate a more physiologically complex ICM (eg, [40,41]) and is still faster than real time.

#### Strengths and Weaknesses of the Unidirectionally Coupled Complex Model Approach

Increasing model complexity by resolving multiple interconnected IC compartments and autoregulatory feedback mechanisms offers physiological fidelity at the expense of strong parameter dependence and lengthy calculation time. Poor identifiability of dynamically balanced and representative ICM parameters required ad hoc nICP adjustment to obtain realistic results, but these were insufficient for multihour simulations. Additional inferences and/or data are required for practical applications. The nICP estimates, subject to additional posterior modification, qualitatively matched the observed ICP and still lacked realistic ICP pulse amplitude, as previously noted by Wang et al [24]. Although this type of resolution has a high clinical diagnostic value [42-44], it is too computationally expensive for simulations of multiple hours. The use of nonpulsatile (eg, mean) ABP inflow increases computational overhead significantly, but model utility is still precluded by the need for ICM parameter estimation. Further exploration of model #3 is needed to evaluate the clinical diagnostic value of simulations driven by mean ABP over multihour periods.

## Discussion

### Summary

This study compared multicomponent modeling approaches to nICP estimation using commonly available data over multihour timescales to produce actionable clinical information. The purpose was to better inform the direction of estimation development by identifying the advantages, limits, and additional requirements of the 2 options. The choices were to integrate a simple ICM into a systemic hemodynamic model or to unidirectionally couple a more complex ICM to the hemodynamic model component. We assessed these methods based on error (*r*_1_), clinical accuracy (*r*_2_), and speed (*r*_3_) of their estimates as well as on their dependence on data and parameter identification. The first key result is that the bidirectional coupling of the simple model is sufficiently fast and potentially accurate and can be implemented using commonly available q1m ABP data without patient-specific waveforms. Specifically, analysis of model performance during a slow ICH event revealed that inclusion of bidirectional coupling improved the low-frequency model resolution of ICP, improving estimation quality while remaining an order of magnitude faster than real time. The second main result is that the complex model approach is too slow for use in the targeted applications. In particular, model #3 required nearly 6 hours to perform a 1-hour simulation along with ad hoc changes to both input parameters and output solution, which can only be eliminated by parameter estimation from additional input data at additional computation time. Limited by publicly available data, the three model approaches considered here represent practical implementations of existing methods; therefore, this study is a comparison of existing models implemented in a typical, sparse data environment.

The stronger-performing simple model approach may use ABP summary data without patient specificity of the inflow waveform and is able to resolve minute-scale nICP variations at additional costs. Its ICM, originally designed to run on high-frequency joint ABP-CBF samples, was coupled to a hemodynamic model of upstream vasculature derived from the complex model to establish ABP-only data dependence. Our experiments show that simple model data dependence can be further reduced to coarse clinical summary data of phase pressures and heart rate, which is independent of the patient-specific postsystole waveform shape. The use of q1m summary data also serves to filter the aortic forcing, which is an important consideration given that the bidirectional setup is more prone to feedback instabilities originating from inflow noise. Furthermore, summary ABPs are less noisy and therefore reduce spurious feedback instabilities in fully coupled simple models (Figure 7 near 175 minutes).

Slow model speed and the need for ICM parameter identification limit the utility of the complex model. The estimation of nICP under model #3 is an order of magnitude slower than the clock time under pulsatile aortic inflow and is only slightly faster than real time under mean ABP. In both cases, strong parameter dependence renders model initialization difficult, and nICP estimates are inaccurate without posterior modification. Some ICM parameters may not be stationary over multihour timescales and may explain the difficulty in maintaining nondivergent behavior beyond the first hour of simulation. The inference necessary to identify these parameters results in additional computational overhead, making near real-time estimation an unrealistic expectation. However, these parameters provide extremely useful diagnostic information and make complex model estimations more suitable for retrospective analysis rather than operational support.

The main results of this work are summarized below:

1. The inclusion of feedback between ICM and AN-CoW components improves the tracking of higher-order trends over multihour timescales. The bidirectionally coupled single-compartment model #2 features a more accurate resolution of low-frequency ICP components than the unidirectionally coupled model at a lower computational cost than model #3.
2. The nICP estimates using q1m ABP data projected onto pulsatile waveforms are qualitatively similar to those obtained using high-frequency APB data. However, q1m summary data must include the heart rate in addition to diastolic and systolic pressures. This result broadens the applicability of simple models, as summary ABP data are more commonly and promptly available in a clinical setting.
3. Patient-specific waveforms are *not* required to use q1m ABP as simple model inflow data; the quality of nICP depends neither numerically nor empirically on resolving postsystole components of patient waveforms. Therefore, simple models do not require supplemental waveform-resolving data to use the q1m summary ABP.
4. Model #2 has a stronger potential for multihour applications because it does not require any parameters, can be run using commonly available data, and runs approximately seven times faster than real time. This makes it a suitable base for ongoing development, even if additional inference or control is required for practical use.
5. The large number of parameters within the complex, nonlinear ICM of model #3 experience difficult identifiability, and poorly specified parameters lead to divergent or unrealistic behavior. It cannot be adequately configured from available data for stable, multihour simulations and performs significantly slower than real time. This model requires sophisticated inference because its parameters, some of which may be nonstationary, need to be accurately specified.
6. The temporal resolution of model #3 was inherited from aortic inflow. With pulsatile inflow, nICP waveforms are resolved and data-optimized results can be used to characterize autoregulatory and adaptive capacity in retrospective studies. Quasi-operational nICP estimation is possible with a significant a priori investment of time for parameter estimation but only under nonpulsatile forcing where the nICP pulse is not resolved.

### Overcoming Model Limitations

#### Refinement and Assimilation

The presented models have inherent limitations that are not fully realized, and a combination of parameter inference and/or data assimilation together with model improvements are necessary to meaningfully simulate clinically important scenarios. The need for accurate parameters in model #3 is evident, and the slow model speed retards this process. Although the simple bidirectional model (#2) is a strong candidate to build upon, it fails to accurately track the ICP trend and variability of patients with IC hemorrhage or stroke. The presence of raw ABP noise and large waveform variance may also play a confounding role in this limitation. However, failure likely results from omitting CBF data, which are independent of ABP, as well as the lack of parameters and simplified mechanism in the ICM that may not account for underlying IC physiological changes. For example, models #1 and #2 do not parameterize IC volume or impose upper bounds on IC compliance to reflect the thresholds of cerebral autoregulatory processes or other exhausted adaptability. Further limitations of all models include inability to account for many important aspects crucial to the clinical decision-making process, including patient age and other diagnoses; injury mechanism; imaging findings; or treatments such as sedation, neuromuscular blockade, osmolar therapy, and ventilation strategy.

**Figure 8:**
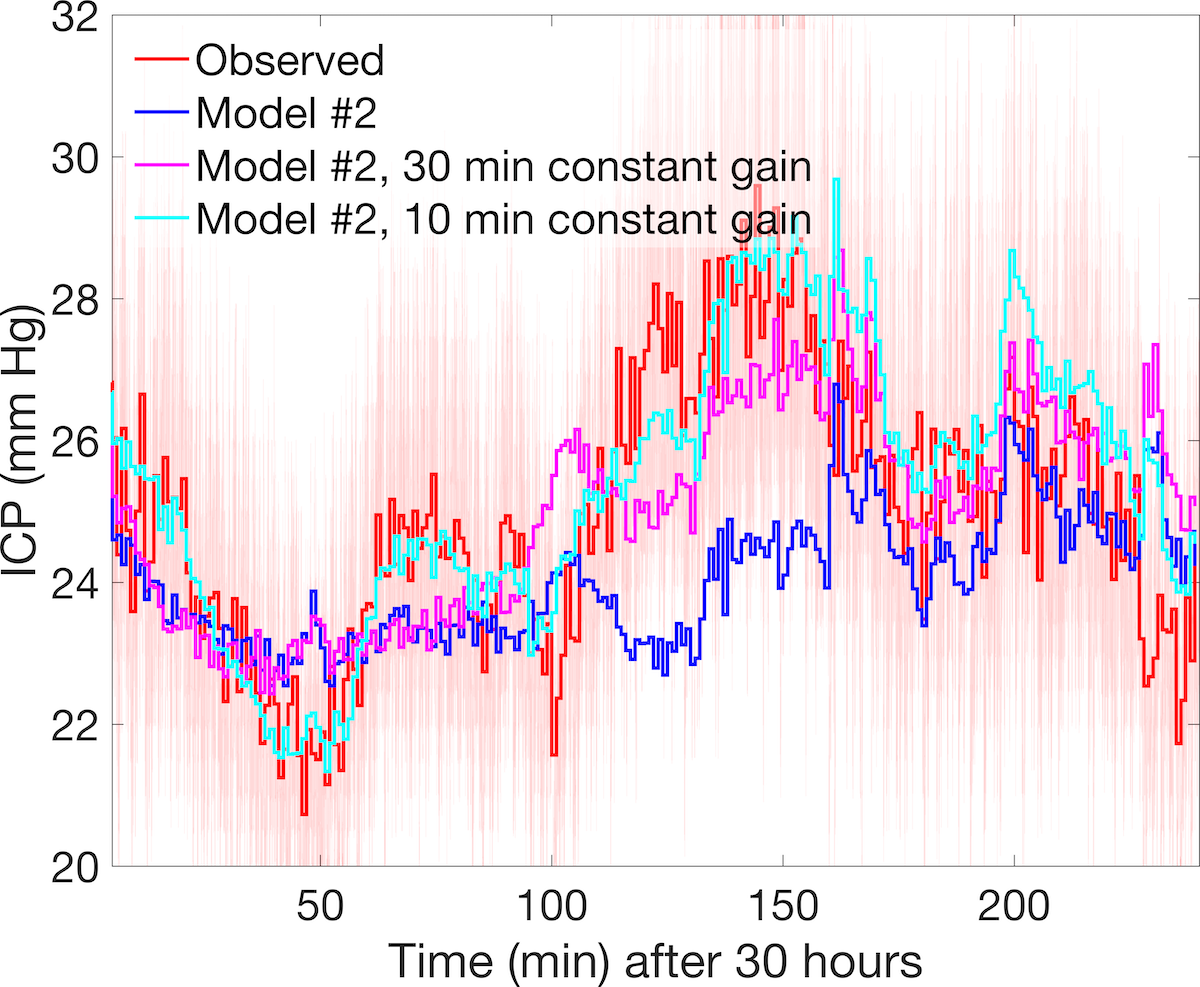
The ICP record for Charis patient #5 during hours 30–34 is shown in light red with its minute-to-minute mean traced in dark red. The observed signal includes stronger signal noise and high-frequency variability than that of patient #6. Slow wave pressure dynamics are observed, but they are absent from the model #2 solution (blue curve), which fails to track the rise and peak of the 7 mm Hg intracranial hypertensive event observed over 100–180 min. The solution using external inflow control specified at 10-minute intervals (cyan curve, using 24 independent parameters) features greatly improved trend tracking during these more dynamic regimes than the solution using parameters specified at 30-minute intervals (magenta curve, using 8 independent parameters). ICP: intracranial pressure.

Overcoming model #2 limitations to estimate nICP for some patients may require a more complex ICM or inclusion of additional dynamically controlled parameters. Many patients of clinical concern, like other Charis patients, have more complicated injuries, and their observed ICP occupies different dynamical regimes than those of patient #6 discussed above. For example, a critical hypertensive period is evident for patient #5, a 21-year-old female with TBI and identified subdural hematoma, whose ICP increased from 21 mm Hg to 29 mm Hg over a 47-minute period (Figure 8, red line) before gradually subsiding. For this patient, the local variability of q1m ICP relative to its 11-minute moving average is about 4 times larger than that of patient #6 (Multimedia Appendix 4). This increased variability is also present in the observed ABP serving as model inflow and may confound both the accuracy and stability of the model. The estimation here benefits from optimized scaling parameters, but additional machinery is necessary to drive model dynamics beyond its inherent ability to predict nICP from ABP. For the example above, the model #2 solution (blue line), using an optimized set of vascular parameters (θ*_l_*, θ*_r_*, ω*_l_*, ω*_r_*, *R_term_*)=(0.8, 1.0, 0.84, 0.93, 1.0) fails to follow the observed dynamics during the central ICH event and sequence of waves leading up to it. Two possible directions for ongoing research—increased fidelity and external parametric control—to improve the performance within the modeling framework are presented.

#### Increased Sophistication

A simple model of increased complexity may account for changes in ICP arising from IC mechanisms, widening the applicability of the framework of model #2. To broaden the scope of potentially modelable cases, other lumped parameter ICMs that offer both increased physiological fidelity and low computational overhead may be considered.

In particular, Ursino and Lodi [41] and Czosnyka and Pickard [43] presented two simple models that offer increased IC process resolution and relevant internal parametrization. Both are directly representable within the electrical analog framework electrical circuit forms [45] and account for elements of autoregulation, varying volumes, and other pressure sources. Either may easily fit bidirectionally within the existing framework as alternate ICM components with sufficiently fast algorithms for the predictive desire discussed above. These models, specifically variations thereof, using the statistical simplification of Kashif et al [15], are part of continuing development within the general purview of this research.

#### Additional Parametrization

Another method of applying the existing simple model #2 to complex cases involves augmented boundary control as a proxy for unresolved processes within a statistical parameter estimation scheme. Although patient-specific optimization is beyond the scope of this study, additional experiments applying the model to ABP-ICP time series of interest show that model #2 is sufficiently robust to track ICP throughout these complex regimes. This requires the addition of modulation of the relationship between ABP inflow at the aorta and the ICM inflow from the MCA using a low-frequency nonstationary gain parameter *G* to vary ABP inflow: ABP(*t*) ← ABP(*t*)·(1+*G*(*t*)). Figure 8 illustrates the potential of this approach by including 2 additional model #2 simulations using 8 and 24 equally spaced control parameters that define *G* piecewise to linearly vary the ABP inflow signal.

The simulation using eight additional parameters (cyan curve) is more dynamic than the base model; it resolves a portion of the central ICH event and decreases the mean error (*r*_1_) by more than 20% (from 8.75 mm Hg to 6.844 mm Hg) but misses its onset and underestimates peak pressure by approximately 1.5 mm Hg. Using 24 additional parameters (magenta curve) further improved this result, improving *r*_1_ by 36% (to 5.633 mm Hg) and identifying the rising trend during the ICH onset as well as its maximum pressure. Determining the values of gain *G* involves placing the current ABP-to-nICP model into a data assimilation system, which provides a meaningful way of automatically constraining uncertainties due to inaccurate parameters and unresolved physiology. Such systems require extensive computational overhead, although some methods such as empirical (ie, ensemble) Kalman-type methods maintain operational estimation of faster-than-real-time models via parallelization. Practical applications require estimation of the parameters defining *G*, although they were specified a priori in this illustration, but underscore the need for simple, fast models to meet the goal of providing timely, relevant nICP estimation over multihour timescales.

### Forecast Potential for Clinical Support

Bidirectional model #2 provides a basis for analyzing latent empirical relationships among patient signals and model parameters, including trends and covariances, which may be used to predict patient ICP changes. Such a system would greatly benefit both clinical decision support and care-level logistics by indicating possible changes in patient status with sufficient lead time to adjust room, equipment, and staff. This may also give practitioners advance warning with a timeframe for planning treatments, permitting earlier and lower-risk interventions to combat IC hypertension. Recent works [46-49] include machine learning approaches to ABP prediction and could be used in conjunction with the presented methods for short-term prediction of nICP. The application of these algorithms to low-sample-rate q1m ABP records has not been reported in the literature.

The speed of model #2 indicates that it is a plausible candidate for use within a statistical estimation and forecast scheme that requires many forward model integrations. The accurately identified parameters, together with acceptable simulation speed, add the possibility of practical forecast capabilities based on trends in diagnostically computed model parameters. For the applications discussed in this work, distributional trends and higher-order moments in ICM resistance and compliance may be inferred from robustly optimized model #2 simulations of a patient’s relevant history. This statistical information may then be used to predict possible future ICP outcomes under current ABP measurements or ABP forecasts, potentially providing valuable and timely clinical decision support for caretakers and facility management.

### Conclusions and Ongoing Work

This study identified the distinct advantages and disadvantages of the 2 paths within a modeling framework and clarified the applicability of each. Although model #2 was more successfully validated at multihour timescales, it required uninterpretable control parameters (*G*) in more complex cases. In contrast, the ICM of model #3 is highly parameter dependent and difficult to identify from accessible data, even for simple cases. These results ultimately motivate the development of a hybrid approach that strategically combines simplifications of the mechanistically resolved processes of model #3 with the speed advantages of locally stationary parameters in model #2. The desire to have an appropriate number of physiologically interpretable parameters for data-optimized modeling contextualizes the problem as one of mechanistic machine learning [50-52].

Our preliminary hypothesis of this work was that the high degree of anatomical fidelity offered by the complex multicompartment model would provide the most diagnostic information from available data. It also had numerous model parameters that could be inferred from patient data in the longer view of the research program, which is to aid in patient-specific clinical support. We pursued an implementation of model #4 using the spatially-resolved vascular system and complex ICM [23], which had recently been used within a data assimilation system [24]. Concern for speed motivated the elimination of the spatial resolution of vessels within the hemodynamic model by adopting the 0D electrical framework, but this approach could not be easily bidirectionally coupled to the analytical ICM. It remained unidirectionally coupled and became model #3. In contrast, the simple model (#1, [15]) was easily integrated bidirectionally into the AN-CoW system, becoming model #2, and this eliminated its dependence on localized CBF data. This fully incorporated model had better tracking of lower-frequency trends in ICP and could resolve higher-frequency ICP waves with additional computational cost, and importantly, it did not require the additional parameter identification of the offline complex ICM for simple cases. However, the lack of sophistication and parametrization in models #1 and #2 is the reason why external parameters for additional control are required for more complex patient cases.

Although the need for additional inference is clear for the application of models #2 and #3, there are substantive differences in methodology and potential benefits. Namely, simple model #2 is easily identified but is limited to applications where a strong correlation between systemic ABP and ICP response is present. This lack of internal parameters necessitates the use of nonstationary external controls for application in complex cases. Although these parameters may plausibly be estimated via ensemble filtering, they are not interpretable, and the necessary mapping between clinical data and the control parameters is unknown and requires further development. In contrast, model #3 has numerous highly interpretable and diagnostically informative parameters that must be properly inferred for meaningful simulations. These are likely estimable from historic patient data using traditional methods (eg, MCMC estimation or optimization), but the value of this investment may be limited if parameters are dynamic and/or only nICP estimation is sought. Given that the estimation of nICP, rather than clinical interpretability, is the primary objective of this project, the continued development of inference machinery for model #2 is the best choice.

The long-term vision of this project remains the development of a bidirectionally coupled model with anatomical fidelity (ie, model #4) fast enough for pre-emptive diagnostic uses such as nICP forecast and the identification of pathophysiology. One path toward this goal is the hybridization of methodologies that integrate an ICM of intermediate complexity under piecewise stationarity assumptions akin to those of simple models. Possible ICMs include those mentioned previously and a simplified (eg, linearized) counterpart of model #3. This should reduce the computational burden of the complex model and allow it to be more easily coupled interactively with the upstream vascular component. Such a model would further benefit from highly interpretable inference based on data available when administering care, with the additional advantage of supporting summary ABP inflow. A remaining question is whether a model formulated in this way can be made fast enough to provide timely and clinically actionable information.

## Appendix

Multimedia Appendix 1 Detailed vessel-level parameterization of the hemodynamic model.

Multimedia Appendix 2 Description of the 6-compartment intracranial model, including tables of parameters and variables and a description of the iterative numerical scheme used.

Multimedia Appendix 3 Description of the single compartment intracranial model.

Multimedia Appendix 4 Supplemental figure.

Multimedia Appendix 5 Explicit description of the parametric waveform model used to transform the arterial blood pressure summary data to a continuous waveform.

Multimedia Appendix 6 Supplemental table.

## Abbreviations

ABP: arterial blood pressure
AN: arterial network
CBF: cerebral blood flow
CoW: Circle of Willis
CPP: cerebral perfusion pressure
CSF: cerebrospinal fluid
IC: intracranial
ICH: intracranial hypertension
ICM: intracranial model
ICP: intracranial pressure
MCA: middle cerebral artery
nICP: noninvasive intracranial pressure
q1m: quaque 1-minute
TBI: traumatic brain injury
