# Supplementary material for: Clinical Decision Support for Traumatic Brain Injury: Identifying a Framework for Practical Model-Based Intracranial Pressure Estimation at Multihour Timescales
Source: JMIR Med Inform. 2021 Mar 22;9(3):e23215. doi: 10.2196/23215 (PMC8077603; doi:10.2196/23215)
Supplement: Multimedia Appendix 1 [file medinform_v9i3e23215_app1.pdf]

# Vessel-level parametriation of the hemodynamic model

The AN-CoW model is comprised of a subcranial arterial network and the CoW vessels, which are modeled using 3-element electrical analogs in MatLab SimuLink. Within them, vessel state variables pressure $P$ and flow $Q$ evolve (as voltage and current, respectively) under the influence of local vessel parameters $(R, C, L)$ and states of adjoining vessels. Base values for all vessel-level parameters and boundary conditions were adopted from previous studies (*viz.* [**?**] and references therein).

**Vessel-level parametrization in the AN-CoW** Physical parametrization of vessel-level hemodynamics in the AN-CoW involves vessel dimensions (cross-sectional area $A_0$ and length $l$), material properties (vessel linear compliance $\partial P/\partial A$, vessel elasticity constant $\beta$, and blood density $\rho$), and a friction scaling term ($\chi$, which depends on vessel mechanical properties and flow profile [**?**]). A local elastic pressure model $P = P(A; \beta, A_0)$ is adopted from [**?**], where

$$\Delta P = \beta / \left( A_0^{-1/2} - A^{-1/2} \right) \tag{1}$$

is the change in internal pressure with respect to transmural vessel pressure. Parameters defining the passive electrical components of each vessels are resistance $R$, capacitance $C$, and inductance $L$. These may be define approximated [**?**] from the physical parameters according to

$$R = \frac{\rho\chi}{A_0^2} \cdot l \tag{2}$$

$$C = 2\frac{A_0^{3/2}}{\beta} \cdot l \tag{3}$$

$$L = \frac{\rho}{A_0} \cdot l \tag{4}$$

respectively. The relationship between $A$ and vessel radius $r$ is elementary.

**Boundary conditions:** Boundary conditions representing unresolved downstream vasculature are 3-element Windkessel (*i.e.* RCR-circuit) models. Outflow boundaries at the cerebral arteries are by defined current ICM pressure and a resistances. These resistances are set as in [**?**] so that bilateral arterial

flows intially target 1.3 ml/s, 2.2 ml/s, and 1.15 ml/s across anterior, middle, and posterior cerebral arteries, respectively, under initial ICP of 15 mm Hg.

In bi-directionally coupled models, CoW terminal vessels connect directly with the necessary IC vessels and require ICM pressure and resistance to coordinate with AN-CoW outflow. Specifically, the currently known estimates of pressure and resistance within the ICM are applied to the bi-directionally coupled middle cerebral artery. Remaining uncoupled CoW termini are set as in the uni-directionally coupled case.

**Parameter Reduction:** We assumed that vessel length and radius dimensions $(l, r)$ scale uniformly within the AN and globally parametrized LRC values according to proportionalities $(\theta_l, \theta_r)$ in relation to the base values. This defines a nonlinear transformation of the electrical parameters via $(R, C, L) \leftarrow \theta_l \cdot \left(\theta_r^{-4} R,\, \theta_r^{3} C,\, \theta_r^{-2} L\right)$. The three remaining parameters – those for 3-element Windkessel boundaries and CoW outflow resistance – are handled analogously with proportions $(\omega_l, \omega_r)$ and $R_{term}$, respectively. Because CoW and adjacent vessel radii are approximately adult-sized by about 5 years of age [**?**], we did not scale vessels within the CoW model component.

Scaling the reference values *en masse* is effective within a realistic range of parameter values, as shown in Fig 3 of the main text. This figure summarizes the relative effect of scaling parameters on properties of ICM inflow signals, determined by 500 simulations of parameters uniformly sampled from 0.5–1.5 for lengths and resistance and 0.9–1.1 for radii. Properties of ICM inflow signals are most sensitive to scaling of AN vessel dimensions and are less sensitive to scaling of the terminal resistance and AN boundary Windkessel values. Scaling of AN vessel dimensions is more influential on the ICM than those related to CoW terminal resistance and AN boundary Windkessel values. This re-parametrization reduces the AN-CoW component identification to five proportionalities $(\theta_l,\, \theta_r,\, \omega_l,\, \omega_r,\, R_{term})$. It establishes a simple system-wide control over the vascular properties, improves parameter sensitivity, and provides a meaningful path to accurate model identification.

Figure 1: **Monte Carlo sensitivity experiments:** Monte Carlo sensitivity experiments performed on the base AN-CoW components with fixed ICP and IC parameters shows how scaling parameters (in columns, at bottom) affect IC component boundary forcing. Each 1-minute simulation (blue points) used artificial ABP forcing in the form of a 1Hz cycle comprised of a 0.15 second sinusoidal systolic upswing to 125 mm Hg followed by a 0.15 second return to 80 mm Hg diastole. The top three rows correspond to values of the mean, variance, and maximum of MCA pressure, and the bottom rows correspond to MCA flow. Columns, left to right, correspond to scale parameters for vessel length, vessel radius, Windkessel length, and Windkessel radius. Red lines establish the relationship between changes in scale parameters and the response in MCA signal properties; their slopes are used in the regression ranking (Fig 3 of the main text). Random joint variations of scale parameters (and terminal resistance scaling, not shown) are sampled and assigned to 500 simulations via Latin hypercube sampling. Uniform sampling distribution ranges were $[0.5, 1.1]$ for lengths, $[0.9, 1.1]$ for radii, and $[0.5, 2]$ for resistance, with weak and positive covariances (0.5) assumed between lengths (and radii) in an attempt to preserve anatomical fidelity.
