# Supplementary material for: Clinical Decision Support for Traumatic Brain Injury: Identifying a Framework for Practical Model-Based Intracranial Pressure Estimation at Multihour Timescales
Source: JMIR Med Inform. 2021 Mar 22;9(3):e23215. doi: 10.2196/23215 (PMC8077603; doi:10.2196/23215)
Supplement: Multimedia Appendix 2 [file medinform_v9i3e23215_app2.pdf]

Description of the 6-compartment intracranial model

The six-compartment model [1] is computationally centered at the the distal cerebral arterial bed represented by the complaint structures C_{d_i} of each of the 6 territories visible in Fig 2 of the main text. The physiological model combines Laplace's for law balancing wall tension with arterial pressure. Representations for tension as functions of pressure P gives:

$$P_d = P_e - \frac{Q_d}{2G_d} \tag{1}$$

$$P_d r_d - P_{ic}\left(r_d + h_d\right) = T_e + T_v + T_m \tag{2}$$

$$T_e = h_d \cdot \left(\sigma_{e0}\left[\exp\left(K_\sigma \frac{r_d - r_{d0}}{r_{d0}}\right) - 1\right] - \sigma_{coll}\right) \tag{3}$$

$$T_v = \frac{h_d \eta}{r_{v0}} \cdot \frac{dr_d}{dt} \tag{4}$$

$$T_m = T_0(1+M)\exp\left(-\left|\frac{r_d - r_m}{r_t - r_m}\right|^{n_m}\right) \tag{5}$$

where subscripts e, d correspond to the proximal and distal arterial beds. Values of P_e correspond to interface pressure between the vascular network and the ICM at CoW outflows, whose character varies depending on the coupling method. Values of Q_d and ΔQ_{coll} represent flows determined by transported fluid balances of each cerebral compartment.

The tension term T_m models autoregulation through modulation of the state-dependent variable $M \in [-1, 1]$ determining vaso-dilation/constriction of effective vascular radius r_d of each compartment. Autoregulation is modeled by the dynamics of a feedback mechanism ξ that aims to relax the distal lumped flow Q_d to a target flow Q_n over timescale t_{CA} with gain factor; the adjustment ODE determines M as:

$$t_{CA}\frac{d\xi}{dt} = -\xi + K_{CA}\frac{Q_d - Q_n}{Q_n} \tag{6}$$

$$M = \frac{e^{2\xi} - 1}{e^{2\xi} + 1} \tag{7}$$

The volume balance for each territory is given in terms of its effective vascular radius r_d:

$$\frac{dV_k}{dt} = 2K_v r_{d_k}\frac{dr_{d_k}}{dt} = G_{d_k}\left(P_{e_k} - 2P_{d_k} + P_{e_k}\right) + \Delta Q_{coll_k}, k = 1\ldots 6 \tag{8}$$

where $G_{d_k} = K_{g_k} r_{d_k}^4$.

The collateral flow volumes ΔQ_{coll_k} are determined by pressure-difference-driven flows between adjacent compartments (see Eqs.25 in [1]).

Once blood flow distribution of each compartment is represented, the common ICP value P_{ic} for the component is the solution to the differential equation

$$C_{ic}\frac{dP_{ic}}{dt} = \sum_{k=1}^{6}\left(\frac{dV_k}{dt} + I_{f_k}\right) - I_0 \tag{9}$$

However, the undetermined ICP influences both CSF outflow I_o and the bed-wise CSF production rates I_{f_k} as well as the the intracranial compliance C_{ic}. Eq (9) must therefore be solved with the nonlinear terms (for

$k = 1 \dots 6$)

$$C_{ic} = \left[K_e \left|P_{ic} - P_{icn}\right| + C_m^{-1}\right]^{-1} \tag{10}$$
$$I_o = G_o(P_{ic} - P_s) \cdot [\![P_{ic} > P_s]\!] \tag{11}$$
$$I_f = G_f(P_c - P_{ic}) \cdot [\![P_c > P_{ic}]\!] \tag{12}$$

with double brackets denoting test operators.
Numerics: The system of equations for the model is represented numerically as

$$\begin{cases} \xi^{t+1} & = (1 - \Delta t)\xi^t + \Delta t K_{CA}(Q_{d}^t \cdot / Q_n - 1)) \\ P_{ic}^{t+1} & = P_{ic}^t + \Delta t \left[-I_0(P_{ic}^t) + \sum_{1:6} \left(\frac{dV}{dt}(r_d^t, r_d^{t-1}) - I_f(P_{ic}^t, P_c^t)\right)\right] \end{cases} \tag{13}$$

and solved by minimizing the nonlinear function $R(x) = |M(x)x - b(x)|$ where

$$x = \begin{bmatrix} r_d \\ P_{ic} \\ P_d \\ P_c \end{bmatrix} \qquad b(x) = \begin{bmatrix} \Delta Q_{coll} - P_e(x)G_d(x) \\ T_e + T_m \\ -G_{pv}P_{ic}^{t+1} \\ r_d^t \end{bmatrix} \tag{14}$$

and

$$M(x) = \begin{bmatrix} 2K_v \frac{dr_d}{dt}(x) & 1 & 2G_d(x) & -G_d(x) \\ P_d(x) - P_{ic}^t & \sigma_v + P_{ic}^t & 0 & 0 \\ 0 & 0 & G_d(x) & -G_{pv} - G_d(x) \\ 1 & -\Delta t & 0 & 0 \end{bmatrix} \tag{15}$$

and is initialized using known or computed values at t.

The entry-wise values of the optimum x provide updated values of its constituents at time $t + 1$. The last row of the system enforces a finite difference approximation to dr/dt, but it makes the system explicitly Δt-dependent.

Tables of Parameters

The complex six-compartment ICM involves many parameters which must be supplied *a priori* for simulation are listed in Table 1. The formulation allows for compartment-level specification of variables indicated by asterisks. Diagnostic variables, *i.e.* those calculated during simulation in addition to nICP, are listed in 2.

References

[1] Jaiyoung Ryu, Xiao Hu, and Shawn C Shadden. A coupled lumped-parameter and distributed network model for cerebral pulse-wave hemodynamics. *Journal of Biomechanical Engineering*, 137(10):101009-1–101009-13, 2015.

[2] Mauro Ursino and Carlo Alberto Lodi. Interaction among autoregulation, CO2 reactivity, and intracranial pressure: a mathematical model. *American Journal of Physiology-Heart and Circulatory Physiology*, 274(5):H1715–H1728, 1998.

Table 1: **Required primitive parameters in the six-compartment ICM.**

Symbol	Description	units
G_f	* CSF formation conductance	(ml/s)/mm Hg
G_{pv}	venous bed capillary conductance	(ml/s)/mm Hg
h_d0	lumped distal vessel wall base thickness	cm
σ_{e0}	passive elastic tension scale parameter	mm Hg
K_σ	growth rate of elastic tension with vessel radius	-
r_{d0}	reference vessel radius for T_e	cm
σ_{coll}	maximal negative vessel tension	mm Hg
T_0	* maximum tension for active tension	mm Hg cm
r_m	* maximal force smooth muscle radius [2]	cm
r_t	* "campanular" relationship scale parameter	cm
n_m	* "campanular" relationship shape parameter [2]	-
η	arterial wall viscosity	mm Hg s
t_{CA}	* CA feedback timescale	s
K_{CA}	* CA feedback gain factor	mm Hg^{-1}
Q_n	* CA feedback target flow rate	ml/s
K_e	P_{ic}^{-1}:C_{ic} ratio parameter	ml^{-1}
C_m	C_{ic} bounding parameter	ml/mm Hg
P_{icn}	P_{ic} offset parameter	mm Hg
K_v	Volume:radius gain parameter (ideally π-times-length)	cm
K_g	* territory conductance:radius^4 (*i.e.* G_d/r_d^4) proportion	-
G_{cAA}	anterior distal flow conductance	(ml/s)/mm Hg
G_{cPP}	posterior distal flow conductance	(ml/s)/mm Hg
G_o	CSF outflow conductance	(ml/s)/mm Hg
P_s	sagittal sinus pressure	mm Hg
$G_{[L/R]AM}$	flow conductance between A/M compartments	(ml/s)/mm Hg
$G_{[L/R]MP}$	flow conductance between M/P compartments	(ml/s)/mm Hg

Table 2: **Diagnostic variables in the six-compartment models.**

Symbol	Description	units
C_{ic}	Intracranial compliance	ml/mm Hg
r_d	Representative vessel radius	cm
$Q_{[L/R]AM}$	distal flow between A/M compartments	ml/s
$Q_{[L/R]MP}$	distal flow between M/P compartments	ml/s
Q_{cAA}	anterior distal flow	ml/s
Q_{cPP}	posterior distal flow	ml/s
