# Supplementary material for: Clinical Decision Support for Traumatic Brain Injury: Identifying a Framework for Practical Model-Based Intracranial Pressure Estimation at Multihour Timescales
Source: JMIR Med Inform. 2021 Mar 22;9(3):e23215. doi: 10.2196/23215 (PMC8077603; doi:10.2196/23215)
Supplement: Multimedia Appendix 3 [file medinform_v9i3e23215_app3.pdf]

Description of the single compartment ICM

The single compartment model [1] seeks to identify IC compliance and resistance by regressing features of the forcing waveforms across temporal intervals. In particular, IC inflow Q and P are represented here in electrical analog form (see Fig 2 of the main text, white inset). The principal assumptions are that ICP, IC resistance C, and IC resistance R are constant over the regression interval, in which case the system reduces to an RC circuit with governing equation

$$Q(t) = \frac{P(t) - ICP}{R} + C \cdot \frac{d}{dt} \left(P(t) - ICP \right). \tag{1}$$

During each systolic upswing $\{t_a <= t <= t_b\}$, flow through the resistance is assumed to be small and the entire flow is stored compliantly; therefore, the value of C is estimated by regressing the net inflow volume against the change in pressure during that interval:

$$C \approx \left[P(t_b) - P(t_a)\right]^{\dagger} \cdot \left[\int_{t_a}^{t_b} Q(t)\, dt \right], \tag{2}$$

with $(\cdot)^{\dagger}$ indicating the pseudo-inverse/least-squares matrix. Identification of intervals $\{[t_a, t_b]\}$ proceeds by identifying roughly the times of minimum and maximum applied pressure.

With the ICM inflow decomposed into resistive and capacitive flows, the former flow is calculated using the estimate of C

$$Q_1(t) = Q(t) - C \frac{dP(t)}{dt}. \tag{3}$$

ICP, assumed to be constant over the interval, is the difference between applied pressure $P(t)$ and the pressure lost to forcing resisted flow:

$$ICP = P(t) - R \cdot Q_1(t). \tag{4}$$

Evaluating at pairs of nearby times t_1, t_2 eliminates ICP, and the value of R is determined by regressing the change in pressure against the corresponding change in resistive flow:

$$R \approx \left[Q_1(t_2) - Q_1(t_1)\right]^{\dagger} \left[P(t_2) - P(t_1)\right]. \tag{5}$$

References

[1] Faisal M Kashif, George C Verghese, Vera Novak, Marek Czosnyka, and Thomas Heldt. Model-based noninvasive estimation of intracranial pressure from cerebral blood flow velocity and arterial pressure. *Science Translational Medicine*, 4(129):1–9, 2012.
