# Supplementary figures and images for: Clinical Decision Support for Traumatic Brain Injury: Identifying a Framework for Practical Model-Based Intracranial Pressure Estimation at Multihour Timescales
Source: JMIR Med Inform. 2021 Mar 22;9(3):e23215. doi: 10.2196/23215 (PMC8077603; doi:10.2196/23215)

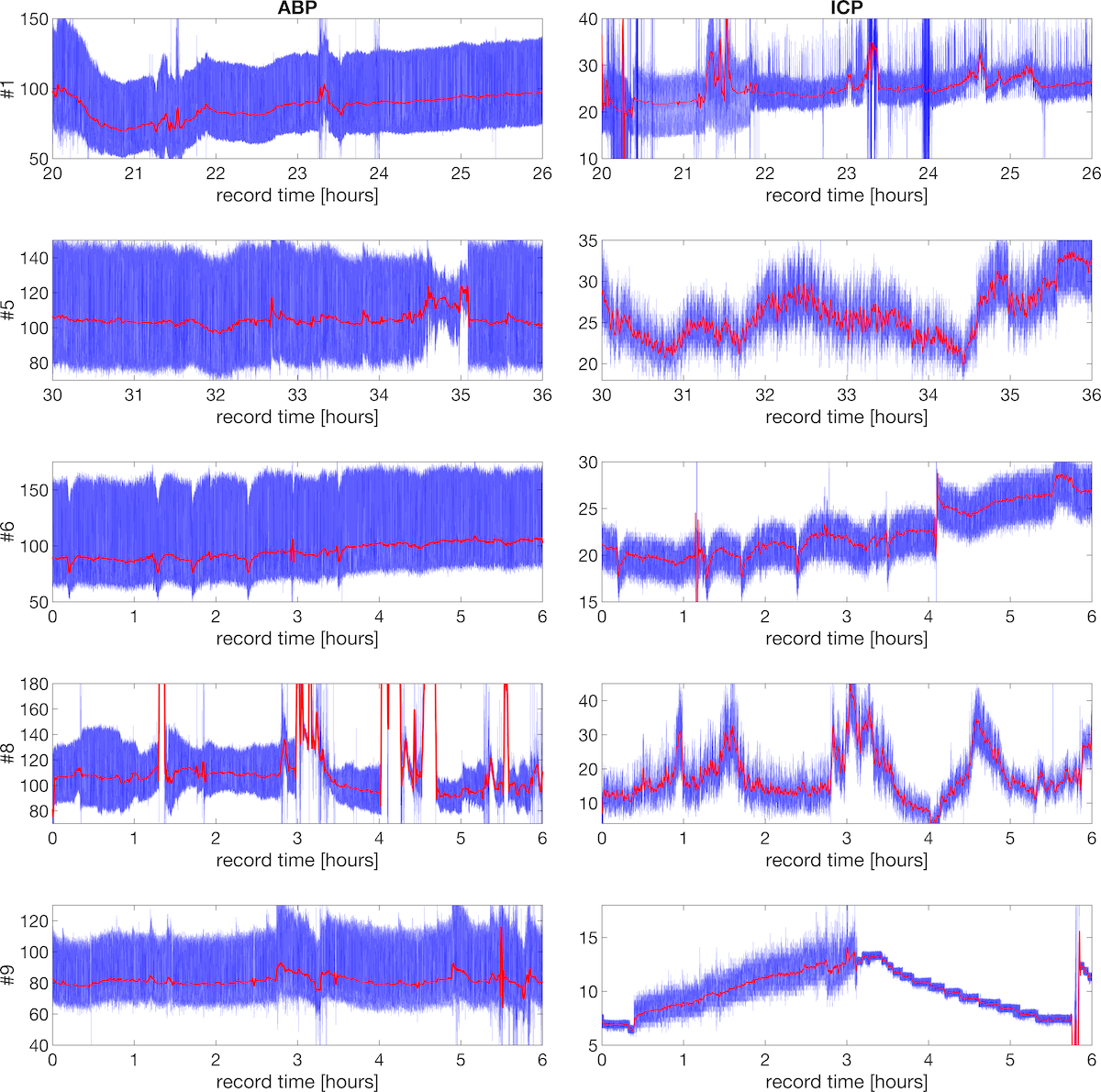

Supplement: Multimedia Appendix 4 [file medinform_v9i3e23215_app4.png]
