# Supplementary material for: Clinical Decision Support for Traumatic Brain Injury: Identifying a Framework for Practical Model-Based Intracranial Pressure Estimation at Multihour Timescales
Source: JMIR Med Inform. 2021 Mar 22;9(3):e23215. doi: 10.2196/23215 (PMC8077603; doi:10.2196/23215)
Supplement: Multimedia Appendix 5 [file medinform_v9i3e23215_app5.pdf]

Transformation of ABP summary data to continuous waveform

Lower-frequency ABP records are assumed to comprise non-overlapping one-minute averages of systolic and diastolic pressures and heart rate (sp,dp, and hr, respectively). In vector timeseries, $X(t_i) = [sp,\ dp,\ hr]$ represents these data during interval i of a *quaque* 1-minute (q1m) record, defining values for $t_i <= t <= t_{i+1}$. Waveform representation is obtained the function

$$\begin{aligned} \widehat{P}\left(t,\ X;\gamma\right) = X_2 + \left(X_1 - X_2\right) \cdot [&\mathrm{Beta}\left(f\left(t,\ X_3\right);\gamma_2,\ \gamma_3\right) \\ &+\gamma_1 \cdot \mathrm{Beta}\left(f\left(1-t,\ X_3\right);\gamma_4,\ \gamma_5\right)] \end{aligned} \quad (1)$$

where Beta denotes the beta distribution probability density function and γ is a static vector of patient-specific waveform shape parameters. The five entries of γ represent the proportion of dicrotic pressure to pulse pressure; beta distribution shape and scale parameters of the systole; and beta distribution shape and scale parameters for the post-systole. Waveform period is modulated to match heartrate via function $f(t,x) = t/x \pmod{1/x}$. The function $\widehat{P}$ transforms q1m information into a piece-wise uniform patient-specific pulsatile signal at an arbitrary resolution suitable for downsampling. Inflow signals constructed thusly from Charis ABP data are conveniently sampled at 60Hz. The patient-specific waveform parameters are trivially estimated from a short (5–10 second) interval of high-frequency observed ABP at negligible computational overhead.
