# Supplementary material for: Clinical Decision Support for Traumatic Brain Injury: Identifying a Framework for Practical Model-Based Intracranial Pressure Estimation at Multihour Timescales
Source: JMIR Med Inform. 2021 Mar 22;9(3):e23215. doi: 10.2196/23215 (PMC8077603; doi:10.2196/23215)
Supplement: Multimedia Appendix 6 [file medinform_v9i3e23215_app6.docx]

|  | Accuracy | Precision | Recall | F1 | Error Rate |
| --- | --- | --- | --- | --- | --- |
| Model #1 | 0.877 (0.92) | **0.980** (0.818) | 0.845 (0.529) | 0.907 (0.642) | 49:309 (1:5) |
| Model #2 | **0.967** (0.98) | 0.944 (0.536) | **0.958** (0.882) | **0.950** (0.667) | **14:165** (1:5) |
| Model #3 | (0.883) | (0.770) | (0.588) | (0.667) | (1:5) |
